# Servelle-Martorell syndrome with extensive upper limb involvement: a case report

**DOI:** 10.1186/1752-1947-2-142

**Published:** 2008-05-03

**Authors:** Raju Karuppal, Rajendran V Raman, Brijesh P Valsalan, TS Gopakumar, Chathoth Meethal Kumaran, Chembu Kara Vasu

**Affiliations:** 1 Medical College, Calicut, 673008 Kerala, India

## Abstract

**Introduction:**

Angio-osteohypotrophic syndrome is also known as Servelle-Martorell angiodysplasia. It is characterized by venous or, rarely, arterial malformations, which may result in limb hypertrophy and bony hypoplasia. Extensive involvement of the upper limb is a rare feature of Servelle-Martorell syndrome. Cases with minimal upper limb involvement have been described in the literature.

**Case presentation:**

A young man presented with multiple separate swollen areas over the right upper limb and functional difficulty since birth. The arm muscles and muscles of the limb girdle were atrophic. The forearm and hand bones were hypoplastic and tender.

**Conclusion:**

We report a case of Servelle-Martorell syndrome with extensive involvement of the entire upper limb and periscapular region. Servelle-Martorell syndrome is highlighted as one of the causes of angiodysplastic limb hypertrophy.

## Introduction

Servelle-Martorell syndrome is characterized by limb hypertrophy owing to venous and rarely, arterial, malformations with skeletal abnormalities (hypoplasia) [1]. Similar conditions such as Klippel-Trenaunay, Parkes-Weber and Blue rubber bleb nevus syndromes can present with limb and bone hypertrophy. MRI is the best imaging method for diagnosis [1]. Adequate radiological investigations with corroborative clinical findings are crucial to establish correct diagnosis. The prognosis of this disorder is uncertain. Therapy is predominantly conservative. In the presence of aneurysmal complications or severe shunting, surgery may be indicated. Servelle-Martorell syndrome has been reported rarely in the literature.

## Case presentation

**Figure 1:**
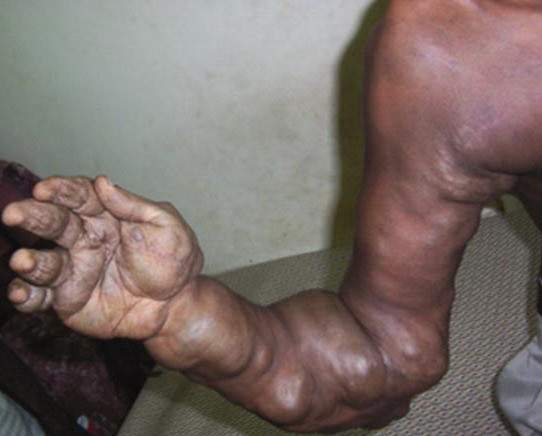
Multiple soft tissue swelling involving the entire upper limb, axilla and periscapular region.

A 21-year-old man presented with an enlarged right upper limb and functional difficulty. The arm had been larger than the opposite limb since birth and was occasionally painful and swollen. The pain and swelling were worse when the limb was lowered. Close examination showed multiple separate swollen areas over the whole of the arm and shoulder girdle (Figure 1). These differed in size; they were soft and compressible, and significantly decreased in size with elevation. The right arm was shorter than the left and this reduction in length was due to overall shortening rather than localized shortening within a particular section of the limb. The arm muscles and muscles of the limb girdle were atrophic.

The palm had a bluish discoloration. Other parts of the body showed no abnormalities. The peripheral pulses were palpable with equal volume on both sides. The forearm and hand bones were hypoplastic and tender. There was no sensory deficit. The muscle strength of the right upper limb was Medical Research Council (MRC) grade III to IV. No bruits or thrills were found. No temperature difference was observed. The elbow flexed fully but had restriction of extension when held in a position of 80 degrees of fixed flexion. The cardiovascular system was normal.

**Figure 2:**
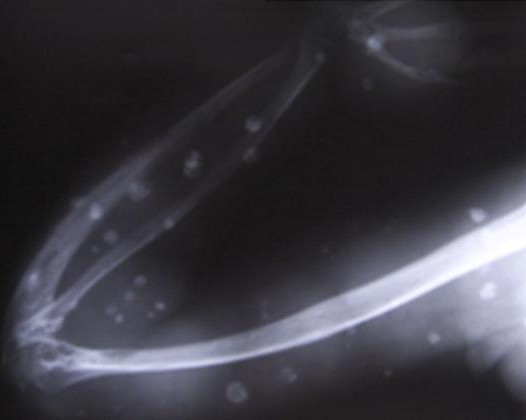
X-ray showing multiple soft-tissue swellings, hypotrophy of the bone, and multiple well-defined, radio-opaque lesions consistent with phleboliths.

Investigation revealed a normal blood picture. Radiographs showed multiple soft tissue swellings and hypotrophy of the bones of right upper limb. There were multiple well-defined radio-opaque lesions consistent with phleboliths in the affected upper limb and periscapular region (Figure 2).

Musculoskeletal ultrasound showed multiple dilated tortuous anechoic lesions involving the upper limb and periscapular region. Echogenic lesions with shadowing suggestive of phleboliths were seen inside the anechoic lesions. The forearm muscles were thinned and replaced by these anechoic lesions.

Color Doppler study showed no flow within the lesion but, while performing a Valsalva maneuver, there was sluggish flow within the lesion suggestive of dilated torturous venous channels involving the superficial venous system. The proximal part of the deep venous system appeared normal but the distal part was not visualized. The arterial system appeared normal.

**Figure 3:**
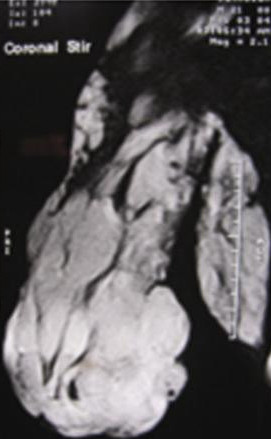
Magnetic resonance imaging scan multiple dilated veins in the superficial aspect of the right upper limb with bone hypoplasia.

An MRI study showed multiple dilated veins in the superficial aspect of the right upper limb (Figure 3). The muscles of the limb were replaced by an abnormal signal. The triceps, biceps and deltoid were partially involved. No intra-osseous venous malformation was seen. The arteries were normal.

We managed this case nonoperatively by external compression with graduated compression stockings. Compression therapy helped to diminish the symptoms of venous insufficiency. The patient does not have any complications such as venous thrombosis, consumption coagulopathy, recurrent cellulitis or recurrent bleeding.

## Discussion

Servelle-Martorell syndrome is also known as phlebectatic osteohypoplastic angiodysplasia [2]. The ectasia and aneurysmal dilatation of the superficial veins may result in a monstrous deformity of the extremity. In the deep venous system, an abnormal vein location, partial or complete lack of valves, and/or venous hypoplasia or aplasia has been observed. Intra-osseous vascular malformations may lead to hypoplasia of the bone with the destruction of spongiosa and cortical bone, resulting in shortening and hypoplasia of the limb [2]. Intra-osseous vascular ectasias can result in joint destruction. Radiographs can demonstrate multiple soft-tissue swellings, hypoplasia of the bones and multiple phleboliths in the venous ectasias. The prognosis of this disorder is uncertain.

Venous vascular malformations span a wide spectrum, varying from isolated cutaneous ectasias to voluminous lesions involving manifold tissues and organs. They are soft and compressible, and show no alteration in skin temperature, thrill or bruits. These are frequently and incorrectly termed 'cavernous hemangiomas'. Pure venous malformations usually exhibit blue coloration of the skin or in the overlying mucosa, while the combined venous malformations and capillaries exhibit a hue that ranges from dark-red to violet [1,3]. The venous malformations are hemodynamically inactive, with a low flow. The condition deteriorates with pregnancy or trauma [4,5]. Absence of overgrowth of the limbs distinguishes it from combined vascular malformations, such as Klippel-Trenaunay syndrome. There may be demineralization, hypoplasia or lytic changes in the underlying bones in up to 71% of cases [4].

Venous thrombosis is a regular complication, and the thrombi may be palpated at the point of pain, but in this case there were no complications related to the venous thrombosis. Another possible complication is the development of consumption coagulopathy due to stasis in the ectatic vascular canals [6]. The possibility of consumption coagulopathy must be investigated prior to undertaking any invasive procedures [3-5].

In the majority of cases, diagnosis is made from the clinical features. A simple radiograph may reveal phleboliths and bone hypoplasia at the age of 2 or 3 years. Magnetic resonance is the best examination to delimit vascular malformation [1].

Based on observations of 47 cases of angiodysplasia of types Parkes-Weber, Klippel-Trenaunay and Servelle-Martorell, Langer et al. [7] demonstrated that differentiation of these three syndromes is possible by taking standard X-rays of the extremities (both sides), which are examined under direct magnification (0.1 – 01 mm). The Weber syndrome should be suspected if bone lengthening is seen in association with loss of substances from the skeleton. In the Klippel-Trenaunay syndrome, the bony lesions do not accompany lengthening. In the Servelle-Martorell syndrome, bony lesions appear together with limb hypertrophy [1,4].

Arteriography and phlebography are required in Servelle-Martorell angiodysplasia to demonstrate the ectatic regions of the involved vessels [8], whereas only phlebography may be needed in Klippel-Trenaunay syndrome.

The majority of the reported cases had a limited area of involvement [2]. The extensive involvement of the entire upper limb and the periscapular region made this case rare.

Nonoperative management is adequate for most patients with Servelle-Martorell syndrome. This includes external compression with graduated compression stockings and garments. Compression therapy can be helpful in protecting the limb, even from minimal trauma that can cause bleeding of the large superficial malformations. It has no effect, however, on the ultimate size of the limb. Patients with significant edema of the lower limbs can be treated with diuretics.

Sclerotherapy with local injection of 95% alcohol or 1% sodium tetradecyl sulphur may be used for small lesions. Surgical resection may then be performed following successful obliteration. The embolization of arteries sustaining the malformation is contraindicated since it may provoke tissue necrosis.

Patients with recurrent attacks of cellulitis may benefit from prophylactic antibiotic therapy. Anticoagulants are indicated after deep vein thrombosis or pulmonary embolus. Patients with recurrent superficial thrombophlebitis frequently require daily administration of aspirin or ibuprofen; however, this may promote problems with bleeding.

Surgery should not be done to improve cosmesis at the expense of function. Aneurysmal complications or severe shunting may be an indication of the requirement for surgery. Surgical excision is the definitive therapy, often rendered impossible, however, by anatomic, esthetic and functional limitations [1,9]. Amputation of a grossly hypertrophied, poorly functioning digit may be necessary but a more proximal foot, hand or limb amputation is rarely required. Symptomatic varicosities or localized venous malformations can be removed in selected patients with good results provided that there is a functioning deep vein system. It should be recognized that complete excision of extensive malformations with debulking procedures is seldom possible.

Debulking procedures can damage venous and lymphatic structures and lead to increased edema of the affected part, scar formation, recurrence, chronic wound infection, and chronic weeping lymphoedema [10].

## Conclusion

Servelle-Martorell syndrome is a rare condition, the diagnosis of which can be confused with Klippel-Trenaunay, Parkes-Weber and blue rubber bleb nevus syndromes. Venous malformations are present in all these conditions; bony hypoplasia is characteristic of Servelle-Martorell syndrome. Although it is rare, extensive limb involvement may be seen in Servelle-Martorell syndrome. MRI is useful in assessing the extent of venous malformations. Conservative treatment is recommended in most cases. Sclerotherapy, with or without surgery, is recommended in cases of functional impairment, even if recurrences are frequent.

## Competing interests

The authors declare that they have no competing interests.

## Authors' contributions

RK contributed to conception and design, acquisition of data, and analysis and interpretation of data. RVR contributed to analysis and interpretation of the investigations. BPV contributed to the acquisition of data and data analysis. TSG contributed to conception and design, and revised the manuscript critically for important intellectual content. CMK contributed by revising and giving final approval of the version to be published. CKV contributed by revising the manuscript critically for important intellectual content. All authors read and approved the final manuscript.

## Consent

Written informed consent was obtained from the patient for publication of this case report and any accompanying images. A copy of the written consent is available for review by the Editor-in-Chief of this journal.
